# *Cis*- and *Trans*-Encoded Small Regulatory RNAs in *Bacillus subtilis*

**DOI:** 10.3390/microorganisms9091865

**Published:** 2021-09-02

**Authors:** Sabine Brantl, Peter Müller

**Affiliations:** AG Bakteriengenetik, Matthias-Schleiden-Institut, Friedrich-Schiller-Universität Jena, D-07743 Jena, Germany; Sabine.Brantl@uni-jena.de

**Keywords:** *Bacillus subtilis*, antisense RNA, sRNA, *cis*-encoded regulatory RNA, *trans*-encoded sRNA, dual-function sRNA, RNA chaperone, Hfq, CsrA, SR1

## Abstract

Small regulatory RNAs (sRNAs) that act by base-pairing are the most abundant posttranscriptional regulators in all three kingdoms of life. Over the past 20 years, a variety of approaches have been employed to discover chromosome-encoded sRNAs in a multitude of bacterial species. However, although largely improved bioinformatics tools are available to predict potential targets of base-pairing sRNAs, it is still challenging to confirm these targets experimentally and to elucidate the mechanisms as well as the physiological role of their sRNA-mediated regulation. Here, we provide an overview of currently known *cis*- and *trans*-encoded sRNAs from *B. subtilis* with known targets and defined regulatory mechanisms and on the potential role of RNA chaperones that are or might be required to facilitate sRNA regulation in this important Gram-positive model organism.

## 1. Introduction

Small regulatory RNAs (sRNAs) that act by base-pairing constitute the main class of posttranscriptional regulators in bacteria. They can be divided into *cis*-encoded and *trans*-encoded sRNAs. Whereas *cis*-encoded sRNAs are transcribed from the same genetic locus in opposite direction to their single target RNA and are, therefore, completely complementary to this target RNA, *trans*-encoded sRNAs are transcribed from a promoter somewhere else on the bacterial chromosome and are only partially complementary to their target RNAs. Therefore, they can only form partial duplexes with their—often multitude of—target RNAs. In both cases, target RNA binding results in inhibition or activation of target gene expression. *Cis*- and *trans*-encoded sRNAs employ a variety of mechanisms of action for posttranscriptional gene regulation [1,2,3] which, eventually, result in altered translation or RNA stability.

A second class of sRNAs acts by protein binding. It comprises the ubiquitous 6S RNA that binds the RNA polymerase by mimicking an open promoter and helps to shift transcription from vegetative to stationary phase promoters (rev. in [4]), sRNAs like CsrB/CsrC that sequester small proteins like CsrA at conserved single-stranded GGA motifs and prevent them from binding to mRNAs where they regulate translation, RNA stability or transcript elongation (rev. in [5]), and plasmid ColE1-encoded Rcd that binds to and enhances the enzymatic activity of tryptophanase to delay cell division until plasmid dimers are resolved [6].

The first *cis*-encoded base-pairing sRNAs, i.e., *bona fide* antisense RNAs, were discovered in *E. coli* plasmids in 1981 and intensively studied as regulators of replication (rev. in [7]), conjugation, and maintenance of plasmids (rev. [1,8]), as well as transposition and transduction (rev. in [1,3]). Only in 2001, two bioinformatics and microarray-based approaches followed by Northern blotting led to the discovery of 14 and 17 *trans*-encoded sRNAs from the *E. coli* chromosome, respectively [9,10]. Since then, in almost all bacterial species, such approaches were used (rev. in [11]), and computer programs for target prediction were established (rev. in [12]). Hitherto-discovered mechanisms of action of base-pairing sRNAs include translation inhibition by induction of structural changes around the RBS (ribosome binding site), direct blocking of the RBS or a ribosome standby site, combined translation inhibition and mRNA decay, degradation, processing, or stabilization of mRNAs, target mRNA trapping, or induction of premature transcription termination at Rho-independent or -dependent transcription terminators (rev. in [3]). In addition, a few antisense RNAs act only *in cis* by transcriptional interference [2], and some plasmid-encoded antisense RNAs prevent the formation of an activator pseudoknot that is required for target mRNA translation [1]. Some of these mechanisms were found both in Gram-negative and Gram-positive bacteria, others only in either of them. However, since many more sRNAs have been investigated to date in Gram-negative species such as *E. coli* or *Salmonella enterica* in comparison to Gram-positive species, it is too early to draw conclusions about the prevalence of certain mechanisms in either of them. The only obvious major difference between sRNA regulation in Gram-negative and Gram-positive bacteria is the requirement of RNA chaperones, which is discussed further below.

Two transcriptomics approaches for the prediction of small RNAs in *B. subtilis* were performed in 2009 and 2010 [13,14], which in total discovered 108 putative *trans*-encoded sRNAs. However, in contrast to Gram-negative bacteria like *E. coli* or *Salmonella enterica* [15], the number of *B. subtilis* sRNAs with identified targets is still very limited. In this review, we summarize our current knowledge on all *cis*- and *trans*-encoded sRNAs from *B. subtilis*, for which targets have been identified and physiological functions elucidated. We do not include protein-binding sRNAs or riboswitches in our review.

## 2. Base-Pairing sRNAs

### 2.1. Cis-Encoded sRNAs (Bona Fide Antisense RNAs) in B. subtilis

**Figure 1:**
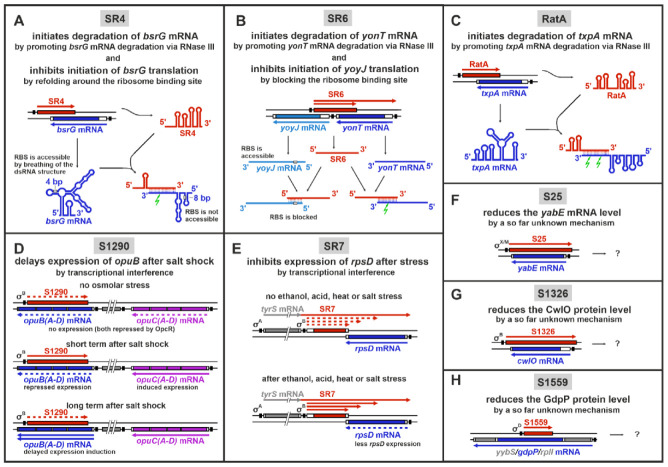
Overview of *cis*-encoded sRNAs in *Bacillus subtilis* and their mechanisms of action. (**A**) SR4 is a bifunctional antitoxin: It promotes the degradation of *bsrG* mRNA and inhibits *bsrG* translation. The SR5/*bsrE* mRNA interaction is highly similar, but SR5 exclusively facilitates degradation of *bsrE* mRNA; (**B**) SR6 represses two toxin genes by two distinct mechanisms: it inhibits the *yoyJ* translation and induces the degradation of *yonT* mRNA; (**C**) RatA promotes *txpA* mRNA degradation; (**D**) S1290 delays the *opuB* expression after salt shock by transcriptional interference; (**E**) SR7 reduces the *rpsD* expression by transcriptional interference under several stress conditions; (**F**) S25 reduces the amount of *yabE* mRNA; (**G**) S1326 represses the *cwlO* expression; (**H**) S1559 represses the *gdpP* expression. The two genomic strands are illustrated as black lines, genes as boxes, and promoter regions as black boxes. The sRNAs and their genes are depicted in red and target genes and mRNAs in blue, other functionally important genes in purple, and unrelated genes in gray; RNaseIII cleavage is represented by a green lightning. In the case of translational inhibition, the RBS is depicted as a grey box.

Currently, 10 systems of *cis*-encoded sRNAs and their corresponding targets are known in *Bacillus subtilis* (see Table 1 and Figure 1), and five of them are type I toxin-antitoxin systems. In total, three mechanisms of target gene regulation were identified: induction of target mRNA degradation, inhibition of translation initiation, and transcriptional interference.

#### 2.1.1. Antitoxins in Type I Toxin–Antitoxin Systems

Toxin–antitoxin (TA) systems are genetic modules comprising a toxin gene and the counteracting antitoxin. These systems are widespread among bacteria and archaea—Both on the chromosome and on plasmids or phages to ensure the stable inheritance of DNA elements. In type I TA systems, the antitoxin is a *cis*-encoded sRNA that interacts with the toxin mRNA. All antitoxins either induce the degradation of the toxin mRNA, inhibit the toxin translation, or combine both mechanisms. The expression of the antitoxin has to be relatively strong to efficiently suppress the toxin expression. The toxins contain typically at least one transmembrane domain and are assumed to act as a small pore-forming protein. So far, only four type I TA systems were investigated in detail: *txpA*/RatA, *bsrG*/SR4, *bsrE*/SR5, and *yonT*/*yoyJ*/SR6 (rev. in [16]). A further 10 potential TA systems were predicted but not yet verified [17].

The *txpA*/RatA system encodes the small toxin TxpA (59 aa) and its antitoxin RatA [18]. In the absence of RatA, TxpA causes cell lysis on agar plates after five days, and *txpA* overexpression drastically impairs growth. The toxin contains a trans-membrane domain followed by charged aa and likely disturbs membrane integrity by pore formation. The antitoxin RatA, encoded on the opposite strand, overlaps the *txpA* transcript by ca. 120 nt at the 3′ end. The *txpA* RNA–RatA complex is a target for RNase III. This role in toxin mRNA degradation makes RNase III essential for *B. subtilis* [19]. Binding of RatA does not influence the *txpA* translation efficiency but exclusively promotes the toxin RNA degradation. The *txpA*/RatA system is located on the skin element, which is excised during the sporulation process and could prevent loss of this region even during a longer period of vegetative growth to preserve the ability to sporulate [18]. Moreover, TxpA plays a role in biofilm formation by elimination of defective and malformed cells [20].

The *bsrG*/SR4 system is encoded on the SPβ prophage region [21]. The toxin BsrG (38 aa) comprises a transmembrane domain and is similar to the N-terminal part of TxpA. BsrG accumulates in the membrane but does not permeabilize it. Instead, it interferes with the cell-envelope synthesis and causes cell-wall defects, membrane invaginations, and an altered cell shape [22]. Deletion of the antitoxin gene *sr4* leads to cell lysis after overnight incubation at 37 °C [21]. The antitoxin SR4 binds to the 3′ end of *bsrG* mRNA, forming a perfect duplex of 123 bp. SR4 is bifunctional: it induces i) RNase III-dependent degradation of the *bsrG* mRNA and ii) a refolding around the RBS (ribosome binding site) that inhibits translation initiation [23]. Both binding kinetics and binding pathway were investigated in-depth, indicating three consecutive interactions of SR4 stem-loops with the corresponding *bsrG* mRNA regions [23]. The initial interaction site of *bsrG* mRNA comprises a YUNR motif in a loop, a sequence previously shown to be able to form a U-turn [24] that facilitates RNA–RNA interactions. Interestingly, the toxin mRNA is rapidly degraded upon heatshock, and both RNases Y and J1 contribute to its degradation [25].

The *bsrE*/SR5 system is located on the prophage-like element P6 in the chromosome [17]. The toxin BsrE (30 aa) only consists of a transmembrane domain and is less toxic than the other type I TA system toxins. The absence of the antitoxin SR5 is not sufficient to induce toxic effects of BsrE. Only overexpression of *bsrE* from a multicopy plasmid causes cell lysis on agar plates and inhibits growth significantly [26]. The antitoxin SR5 encoded on the opposite strand overlaps *bsrE* by 114 nt at the 3′ end. Complex formation of both RNAs leads to degradation of *bsrE* mRNA by RNase III [27]. In contrast to *bsrG*/SR4, no direct translational inhibition of *bsrE* mRNA by SR5 was observed. The interaction pathway between *bsrE* mRNA and SR5 was investigated in detail [27]: the initial contact is formed between two loops that both contain YUNR motifs, enabling a highly efficient and rapid complex formation. The expression of *bsrE* and *sr5* is regulated differently in response to a multitude of stress factors. The otherwise very stable *bsrE* mRNA is sensitive to ethanol and alkaline stress as well as heatshock, while SR5 amounts differ under acid and alkaline stress, iron limitation, and anaerobic conditions. Interestingly, SR5 is rapidly degraded under oxygen deficiency, which allows *bsrE* expression. Therefore, this TA system could play a role in the response of *B. subtilis* to anaerobic conditions [26].

The *yonT*/*yoyJ*/SR6 system is very special, because it contains two toxin genes that are regulated by the same antitoxin. YonT (59 aa) is a very strong toxin, consisting of an N-terminal transmembrane domain and a highly positively charged C-terminal region. The deletion of the antitoxin gene *sr6* causes cell lysis. YoyJ (83 aa) is a very weak toxin with two transmembrane domains. In the absence of SR6, *yoyJ* overexpression is detrimental to the cells. The antitoxin SR6 uses two distinct mechanisms to repress *yonT* and *yoyJ*: SR6 and *yonT* mRNA interact at their 3′ ends inducing RNA degradation and, therefore, toxin inactivation by RNase III [19]. The interaction between SR6 and *yoyJ* mRNA involves both 5′ ends and does not influence the *yoyJ* mRNA stability. Instead, SR6 inhibits the *yoyJ* translation by blocking the RBS [28]. Surprisingly, the *sr6* promoter is much weaker than the *yonT* promoter, which is unfavorable to generate an excess of the antitoxin. However, this is compensated for by an unusually stable antitoxin. The TA system is located on the SPβ prophage region of the chromosome and, like *bsrE*/SR5, both RNAs are impacted by several stress conditions.

An excess of *yonT* mRNA under heatshock or manganese stress suggests a role of the system in the cellular stress response [28].

The *bsrH*/as-bsrH system is a potential type I TA system [14,29]. The toxin BsrH (29 aa) is very similar to BsrE, indicating a common origin. So far, the toxicity of BsrH was not proven experimentally. The *bsrH* mRNA interacts with the antitoxin as-bsrH at the 3′ end and is degraded by RNase J1 [17]. The system is located on the skin element directly adjacent to, but independent of, the *txpA*/RatA system.

#### 2.1.2. Other Cis-Encoded sRNAs in *B. subtilis*

The non-coding RNA S25 is an antisense RNA regulating the gene *yabE*. S25 is transcribed under control of the extracytoplasmic sigma factors σ^M^ and σ^X^ in response to cell envelope stress, e.g., the presence of antibiotics. The amount of *yabE* RNA is reduced during S25 expression, which may be due to RNase III-dependent degradation [30]. The function of YabE is so far unknown, but a potential G5 domain (N-acetylglucosamine binding domain) suggests a function at the cell wall and therefore a role in the response to antibiotics.

The sRNA S1559 is transcribed convergently to the *gdpP* gene on the opposite strand. GdpP is a membrane-localized c-di-AMP specific phosphodiesterase [31], which is involved in the control of sporulation initiation and cell-wall homeostasis. The S1559 promoter is σ^D^-dependent, suggesting a role in or during motility regulation. The expression of S1559 reduces the amount of cellular GdpP but does not influence motility or antibiotic resistance [29]. The mechanism of S1559 action is still elusive.

The antisense RNA S1326 is transcribed from a σ^B^-dependent promoter. The transcript was detected after phosphate stress, is of heterogeneous length, and is complementary to the *cwlO* gene. CwlO is an extracellular D,L-endopeptidase-type autolysin involved in the cell wall synthesis and mainly active in the exponential growth phase [32]. Due to rapid RNase Y-dependent degradation, the *cwlO* transcript is very unstable. How the antisense RNA influences the *cwlO* expression and the biological role of this regulation are so far unknown. The only effect observed was a slightly faster entry into exponential growth after resuspension in fresh medium [33].

Another σ^B^-dependent antisense RNA is S1290, which is complementary to opuB mRNA. OpuB is a choline importer required to maintain the osmotic homeostasis under high salinity conditions. The transiently transcribed S1290 acts by transcriptional interference to repress a fast and strong *opuB* induction after osmotic shock. This delays the *opuB* expression in favor of the expression of *opuC*, which encodes an alternative but more general osmoprotectant import system. Therefore, S1290 is involved in fine-tuning the salt stress response [34].

The sRNA SR7 (S1136) is a dual-function antisense RNA that on the one hand interferes with *rpsD* transcription and on the other hand is an mRNA encoding the small protein SR7P (39 aa). This small protein interacts with the glycolytic enzyme enolase, thereby promoting its recruitment to RNase Y to enhance the RNase Y activity on at least two substrate RNAs, *rpsO* mRNA, and the 5′ UTR of *yitJ* mRNA [35]. Consequently, SR7P plays a role in RNA degradation [35,36]. SR7 only acts *in cis* to reduce the amount of *rpsD* mRNA, most likely by transcriptional interference [37]. RpsD (22.7 kDa) is the primary RNA binding protein S4 of the 30S ribosomal subunit. Therefore, a reduction of the SR4 amount finally decreases the number of active ribosomes. The σ^B^-dependent transcription of *sr7* is strongly induced under ethanol stress [37] as well as salt, acid, heat, and manganese stress conditions [35]. In addition, SR7P affects cell survival under stress conditions, indicating that it might play a specific role in stress response.

For none of the five antisense RNAs were the secondary structures determined or, in case they base-pair with their target RNAs, the binding pathways elucidated.

A number of other potential antisense RNAs were found in the two transcriptomics studies [13,14] but have so far not been confirmed experimentally.

### 2.2. Trans-Encoded sRNAs in B. subtilis

**Figure 2:**
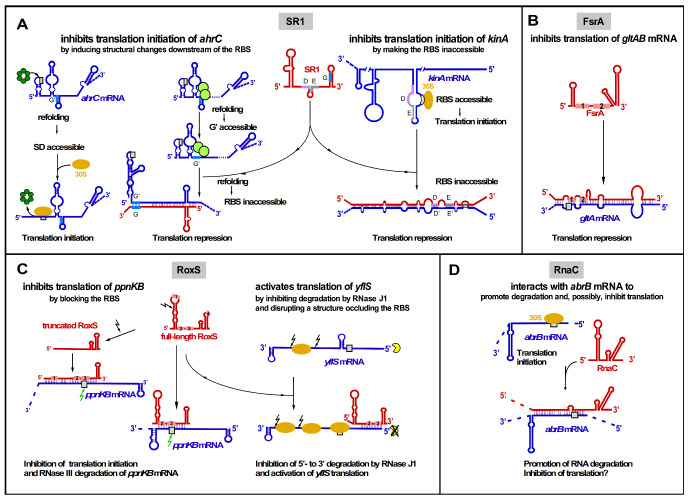
Interactions of the currently known *trans*-encoded sRNAs with their target mRNAs. (**A**) Left half: SR1 interacts with *ahrC* mRNA about 100 nt downstream of the RBS, which induces structural changes around the *ahrC* RBS that inhibit translation initiation. Hfq (dark-green) binds immediately upstream of the *ahrC* RBS (grey rectangle) to make it accessible to the 30S SU. By binding to GGA motives 1–3 of *ahrC* mRNA, CsrA (light-green) induces a slight structural change that makes region G’ (turquois) accessible to complementary region G of SR1. SR1 binding causes a structural change that renders the *ahrC* RBS inaccessible to 30S binding, thereby inhibiting translation initiation [45,46] (based on [4]). Right half: SR1 inhibits translation initiation of *kinA* mRNA by occluding the *kinA* RBS. The initial SR1–*kinA* mRNA interaction occurs between complementary regions D/D’ (violet) followed by the interaction between complementary regions E/E’ (grey). (**B**) Interaction of FsrA with *gltAB* mRNA. FsrA has numerous targets, but in vivo base-pairing has only been shown for *gltAB* mRNA. Most probably, FsrA inhibits translation of all targets (see Table 2). The two C-rich regions (CRR) are highlighted in pink. (**C**) Interaction of RoxS with two target mRNAs. Whereas RoxS and its truncated derivative repress *ppnKB* translation, full-length RoxS activates *yflS* translation, both by disrupting a structure occluding the RBS and by inhibiting RNase J1 degradation from the 5′ end. RoxS interacts with its targets via CRR’s (shown in pink), CRR3 was found to be decisive. (**D**) Interaction of RnaC with *abrB* mRNA, which promotes RNA degradation and, most probably, inhibits *abrB* translation. Red, sRNAs; blue, target mRNAs; beige oval, 30S SU; grey box, RBS; green arrow, RNase III; black arrow, RNase Y; yellow, RNase J1.

At present, five *trans*-encoded sRNAs are known in *B. subtilis* for which targets were identified: SR1 [38,39], FsrA [40], RoxS [41,42], RosA [43], and RnaC [44] (see Figure 2). Among them, SR1 is the only dual-function sRNA that acts as a base-pairing sRNA and as mRNA encoding a small protein, SR1P (see below). In Table 2, an overview of all currently known *trans*-encoded sRNAs in *B. subtilis* and their targets is provided.

#### 2.2.1. SR1

SR1 was the first *trans*-encoded sRNA discovered in *B. subtilis* [38]. A computational approach employed to search for putative sRNAs in intergenic regions of the *B. subtilis* chromosome yielded 20 candidate sRNAs, among them SR1, which was subsequently confirmed by Northern blotting [38]. The *sr1* gene is transcribed under gluconeogenic conditions from the σ^A^-dependent promoter p*_sr1_* and repressed under glycolytic conditions mainly by CcpN and, to a smaller extent, by CcpA [38]. Whereas CcpA binds at a *cre* site about 260 bp upstream of p*_sr1_*, CcpN binds at two sites, directly upstream of the -35 box and in the spacer region of p*_sr1_*, and requires two ligands, ATP and H^+^ (slightly acid pH), for a 20- to 30-fold repression of *sr1* transcription [47,48]. CcpN interacts with the α-subunit of the RNA polymerase to prevent promoter escape [49]. The first identified target of SR1 was *ahrC* mRNA [39], encoding the transcriptional activator of the arginine catabolic operons *rocABC* and *rocDEF* and the transcriptional repressor of the arginine biosynthesis genes [50]. SR1 shares seven complementary regions with *ahrC* mRNA, designated A to G in SR1 and A’ to G’ in *ahrC* mRNA, located in the central portions of both RNAs [39]. Binding starts between region G of SR1 and region G’ 97 nt downstream of the *ahrC* RBS and inhibits translation initiation by a novel mechanism: SR1 induces structural changes 20 to 40 nt downstream of the *ahrC* RBS that obstruct binding of the ribosomal 30S subunit [45].

Although the G/G’ interaction is decisive, the complementary regions B/B’ to F/F’ also contribute to SR1/*ahrC* RNA complex formation [45]. SR1 barely affects the stability of *ahrC* mRNA [39,46], excluding the recruitment of an RNase as a primary mechanism of sRNA action. The RNA chaperone Hfq binds both SR1 and *ahrC* mRNA but does not promote their interaction. Instead, it is required for *ahrC* translation by opening a secondary structure 5′ of the RBS. By contrast, the RNA chaperone CsrA that also binds to both RNAs facilitates their interaction by slightly altering the *ahrC* mRNA structure around the G’ region to support SR1 binding [46] (see Figure 2).

**Figure 3:**
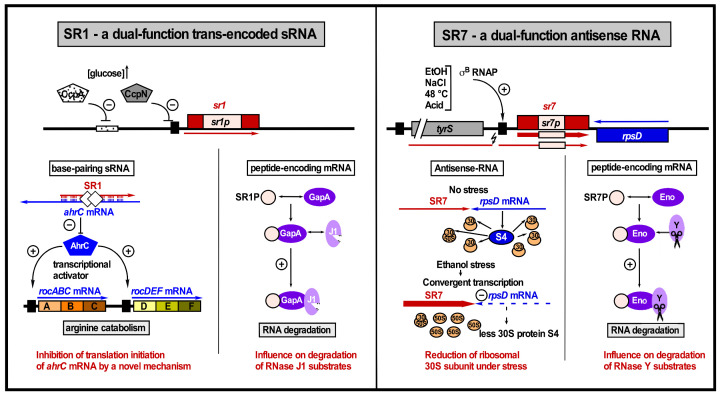
Two dual-function regulatory RNAs from *B. subtilis*, SR1 and SR7. Left: SR1 acts on the one hand as base-pairing sRNA in arginine catabolism by inhibiting translation of *ahrC* mRNA encoding the transcriptional activator of the arginine catabolic operons and on the other hand as mRNA encoding SR1P that modulates the *B. subtilis* DLN (degradosome-like network) by interacting with GapA to promote the GapA-RNase J1 interaction and enhance the RNase J1 activity. Transcription of *sr1* is repressed by CcpA and CcpN. The RNA chaperone CsrA (white diamonds) promotes the SR1-*ahrC* mRNA interaction (adapted with permission from [4]). Right: On the one hand, SR7 reduces by transcriptional interference the amount of the convergently transcribed *rpsD* mRNA and, on the other hand, encodes SR7P that also modulates the DLN by interacting with enolase, increasing the recruitment of RNase Y and enhancing the RNase Y activity. Black line, DNA; red and blue lines, antisense and target RNAs; pink rectangle, SR1P or SR7P ORF.

In addition to being a *trans*-encoded sRNA that acts by a base-pairing mechanism, SR1 is an mRNA that codes for a small protein of 39 aa [51]. This protein, designated SR1P, interacts with the glycolytic GapA (glyceraldehyde-3 phosphatedehydrogenase A, one of the two Gap proteins in *B. subtilis*), promotes the interaction of GapA with RNase J1, and enhances the enzymatic activity of RNase J1 on two substrate RNAs [52]. The SR1P/GapA interaction surface has been elucidated [53]. So far, SR1 is the only dual-function sRNA with functions in different physiological pathways, arginine metabolism, and RNA degradation [36,54] (see Figure 3). An experimental analysis of 9 of the 23 SR1 homologues detected in 2012 demonstrated that both the base-pairing and the protein-encoding functions of SR1 are highly conserved over one billion years of evolution [55]. In the meantime, many more bacterial genomes have been sequenced, and a new search in 2021 yielded 139 SR1/SR1P homologues, all of them confined to the order Bacillales.

Recently, we discovered a second target of SR1, *kinA* mRNA, encoding the major histidine kinase of the sporulation phosphorelay [56]. Upon starvation and stress, KinA autophosphorylates and transfers its phosphate via Spo0F and Spo0B to the central sporulation regulator, the transcription factor Spo0A. Like SR1 and *ahrC* mRNA, SR1 and *kinA* mRNA share seven complementary regions termed A to G in SR1 and A’ to G’ in *kinA* mRNA, the latter including regions upstream and downstream of the RBS as well as within the 5′ part of the ORF. The SR1–*kinA* RNA interaction starts at region D’ 10 nt downstream of the *kinA* RBS and causes translation inhibition, although the RBS itself is not complementary to SR1.

The SR1 D region is located in the central single-stranded part of the molecule, whereas the SR1-*ahrC* mRNA interaction starts within the left arm of the SR1 transcription terminator (see Figure 2A). Similar to the SR1/*ahrC* system, SR1 does not affect the half-life of *kinA* mRNA. Accordingly, SR1P that stimulated the degradation of two RNase J1 substrates via its binding to GapA did not affect *kinA* regulation. Surprisingly, although CsrA binds both SR1 and *kinA* mRNA, it neither promotes their interaction nor affects the regulation of KinA/Spo0A downstream targets or sporulation in vivo. Hfq was also dispensable for SR1-mediated *kinA* regulation and sporulation. Therefore, it cannot be ruled out that another, still unidentified RNA chaperone might fulfil the function of Hfq or CsrA in the SR1/*kinA* system. Interestingly, by controlling *kinA*, SR1 increases the time window for sporulation, allowing the formation of high-quality spores with a proper coat and crust. This function is only required under starvation conditions.

#### 2.2.2. FsrA

The search for additional transcription units regulated by the ferric uptake regulator Fur yielded FsrA, the second small *trans*-encoded *B. subtilis* sRNA, as well as three small basic proteins FbpA, FbpB, and FbpC [40]. The 84 nt long FsrA is a functional homolog of the *E. coli* sRNA RyhB. Iron-deplete conditions lead to derepression of the Fur-dependent *fsrA* promoter and allow FsrA to inhibit translation of target mRNAs involved in iron metabolism and storage. Among the first identified FsrA targets were the succinate dehydrogenase *sdhCAB*, the aconitase *citB* and *lutABC* encoding oxidases important for growth on lactate as sole carbon source.

A transcriptome analysis revealed in 2012 that FsrA is a global regulator with many targets, among them the *gltAB* (glutamate synthase), dicarboxylate transporter (*dctP*), the extracytoplasmic thioreductase *resA*, *leuABCD* (leucine biosynthesis), and menaquinolcytochrome c oxidoreductase *qcrA* genes [57]. On the basis of RNAhybrid and M-fold predictions, the authors suggested that a C-rich single-stranded (CRR) region of FsrA base-pairs with the RBS of its target mRNAs [58]. Hitherto, in vivo base-pairing could be only demonstrated between FsrA and the 5′ UTR of *gltAB* mRNA (Figure 2B). However, mutations that confirmed base-pairing did not involve CRR1 or CRR2, but three complementary regions upstream of them. In addition, EMSAs showed in vitro base-pairing between FsrA and *sdhCAB* mRNA. For the other target mRNAs, effects on mRNA and protein levels were reported, but the predicted base-pairing interactions [40] have yet to be confirmed in vivo to demonstrate that they are primary and not downstream targets of FsrA.

Whereas the functionally related Fur-regulated sRNA RyhB from *E. coli* requires the RNA chaperone Hfq, FsrA cooperates with one, two, or three Fur-regulated small basic proteins FbpA (54 aa), FbpB (48 aa), and FbpC (29 aa) postulated to be RNA chaperones [40]. However, RNA binding has not yet been confirmed for any of them. A concerted action of FsrA and FbpB in the inhibition of *lutABC* mRNA has been shown, which allows the direction of iron to higher priority target proteins [58]. The *lutABC* mRNA levels were affected approximately two-fold by both FsrA and FbpB with FbpB having a higher effect on RNA levels. Only small effects on the LutA, LutB, and LutC protein levels were observed. Whereas FsrA might directly repress *lutABC* translation by a base-pairing interaction, FbpB might facilitate the FsrA-*lutABC* RNA interaction since the role of FbpB could be bypassed by modest upregulation of *fsrA.* Alternatively, FbpB might recruit an RNase for *lutABC* mRNA degradation. So far, experimental evidence for any of these mechanisms is still lacking. For the repression of *sdhCAB* mRNA and *citB* mRNA by FsrA, all three Fbp proteins seem to be dispensable as has been shown by a proteomics study with *fsrA* and *fbp* mutants [58].

#### 2.2.3. RoxS

RoxS (originally termed RsaE, 115 nt) is, so far, the only base-pairing sRNA that is conserved between *B. subtilis* and *S. aureus.* Transcription of *roxS* is activated in response to nitric oxide by the two-component system ResDE [41] and inhibited by the NADH-sensitive transcription repressor Rex [42]. The repression is released by malate. The physiological function of RoxS is to help restore the NAD^+^/NADH balance by temporarily turning down part of the TCA (tricarboxylic acid) cycle. So far, four direct RoxS targets have been experimentally confirmed: *ppnKB* mRNA encoding an NAD^+^/NADH kinase, *sucCD* mRNA encoding succinyl-CoA synthase [41], *yflS* encoding one of four malate transporters transcribed under control of MalKR that senses malate [42], and, recently, *acsA* encoding acetyl CoA synthase [43]. In all cases, RoxS uses several of its four C-rich regions for target binding, among them CRR3 (nt 51-64), which was shown to be decisive. However, RoxS employs different mechanisms of action: It inhibits translation and promotes degradation of *ppnKB* mRNA (Figure 2C) and *sucCD* mRNA. The RoxS–*ppnKB* mRNA duplex is substrate for the double-strand specific RNase III, whereas RNase Y cleaves *ppnKB* mRNA both RoxS-dependently and -independently and RoxS at nt +20, yielding the truncated species RoxS(D). The latter is required for inhibition of *sucCD* translation but can also efficiently regulate *ppnKB*. For *acsA*, fourfold-reduced mRNA levels in the presence of RoxS have been observed [43], but effects on translation were not analyzed. In the case of *yflS*, RoxS disrupts a structure at the 5′ UTR that obstructs ribosome binding to activate translation. Furthermore, it stabilizes *yflS* mRNA about 3.6-fold against 5′-3′ exoribonucleolytic degradation by RNase J1 [42] (Figure 2C). This effect of an sRNA on target mRNA stability is rather an exception, as the majority of bacterial sRNAs examined to date, among them also SR1 and FsrA, directly impact mRNA translation with only an indirect or no effect on the mRNA half-life (rev. in [3]). Translation activation of *yflS* mRNA is independent of its protection against 5′-3′ exoribonucleolytic degradation by RNase J1 [42]. Likewise, the RoxS effects on translation and stability of *sucCD* mRNA can be uncoupled [42].

Similar to SR1 and FsrA, RoxS does not require Hfq, at least for the regulation of *ppnKB* mRNA [41].

#### 2.2.4. RosA

Recently, a sponge RNA, RosA (regulator of sRNA A) was identified that is transcribed from a σ^A^-dependent promoter under control of the central regulator of carbon catabolism, CcpA [43]. Northern blotting revealed four RosA species (225, 193, 128, and 92 nt), the generation of which requires a complex network of different, partially yet unknown, endo- and exoribonucleases. The 225 and the 193 nt species contain three G-rich regions (GRR1-3) with potential complementarity to the C-rich regions of RoxS and FsrA. EMSAs showed that RosA can interact with RoxS and FsrA in vitro. IntaRNA predicted two complementary regions between RosA and RoxS, one comprising 15 consecutive bases involving nt 28-42 (with C-rich region CRR1) of RoxS, the other 10 consecutive bases involving nt 58-68 of RoxS with CCR3 that is decisive for binding of target mRNAs. Mutations in RoxS and RosA species revealed that in vitro, RoxS CRR1 complementary to RosA GRR2 is key to their interaction. In vivo, RosA binding impacts RoxS levels, reduces its half-life fivefold from 30 to 6 min, and promotes processing into the truncated RoxS(D) species. The decreased amount and half-life of three RoxS target RNAs, *ppnKB* mRNA, *acsA* mRNA, and *yrhF* mRNA (a new target RNA) in a Δ*rosA* strain corroborated the hypothesis that RosA sequesters RoxS [43]. Accordingly, an in vivo RNA quantification in *B. subtilis* grown in LB medium yielded a 1:1 RoxS/RosA ratio, indicating that even a complete out-titration of RoxS by RosA would be possible. The RosA effects on RoxS target mRNA levels were substantiated by proteomics confirming altered protein levels for PpnKB, SucC, SucD, AcsA, and YrhF in the absence of RosA. Interestingly, RoxS also plays a role in RosA processing, affecting at least the abundance of the 128 nt RosA species.

Another interesting question raised by the authors is whether the RosA/RoxS duplex might be a reservoir for RoxS or the truncated RoxS(D) species, from which the sRNA can be recycled for base-pairing with new target RNAs for which it has a greater affinity.

The CcpA-dependent RosA regulation places RoxS that is controlled by malate via transcription factor Rex at the key position of central metabolism in response to different carbon sources. The importance of RosA for *B. subtilis* under oxidative respiration conditions was demonstrated by co-cultivation of a wild-type and a Δ*rosA* strain: the Δ*rosA* strain was rapidly outcompeted due to the RoxS- and FsrA-mediated reduction of TCA cycle enzyme levels, which did not allow the mutant to generate ATP as quickly as the wild-type [43].

Why does RoxS, which is regulated by two transcription factors, ResD and Rex, need an additional level of posttranscriptional control by a sponge RNA? The authors argue that the long half-life of 30 min in a Δ*rosA* strain would not allow RoxS to respond quickly to altered environmental conditions and that, therefore, RosA might be required to rapidly downregulate RoxS activity first by neutralizing the C-rich regions required for target RNA binding and afterwards by promoting RoxS degradation [43].

The RosA–FsrA interaction involves RosA GRR2 as well, which binds to CRR2 of FsrA, as also shown in vitro by EMSAs. Proteomics revealed the expected alterations in the amount of proteins encoded by FsrA target mRNAs, among them CitB, SdhA, and CitZ.

Whereas the currently known sponge RNAs from Gram-negative bacteria are either mRNAs [59] or derive from the 3′ UTR of mRNAs [60], a similar stand-alone sRNA sponge named RsaI has been proposed for the staphylococcal RoxS homologue RsaE [61].

RosA contains a seven codon ORF that was associated with ribosomes in a ribosome profiling study [62]. Since the SD for this ORF is located in GRR2, RoxS binding would repress translation of this short ORF.

#### 2.2.5. RnaC

RnaC/S1022 was first identified in a microarray screen of *B. subtilis* intergenic regions. However, so far it has not been visualized in Northern blots. This sRNA is only transcribed during the exponential growth phase under control of σ^D^. Compensatory mutations introduced into predicted complementary loop regions revealed that RnaC base-pairs with *abrB* mRNA at the RBS and the first six codons to modulate the cellular level of transition state regulator AbrB [44] (Figure 3D). The RnaC-*abrB* RNA interaction that did not require Hfq was shown to support degradation of *abrB* mRNA and proposed to inhibit its translation. However, in Δ*rnaC* strains grown in LB medium, only very small alterations (≈33%) of *abrB* mRNA and AbrB levels were detected. The authors observed an enhanced cell-to-cell variation of AbrB levels, which finally resulted in growth rate heterogeneity within one population of logarithmically growing cells. They hypothesized that the subpopulations of fast- and slow-growing *B. subtilis* cells reflect a bet-hedging strategy for enhanced survival of unfavorable conditions since slowly growing cells are less susceptible to harsh environmental conditions [44].

## 3. Conclusions

Although more than 100 sRNAs have been discovered by transcriptome analyses in *B. subtilis* [13,14], and a small number have been found by other approaches [29,38,63], only 10 *cis*-encoded and five *trans*-encoded sRNAs have been characterized in detail. For each of the *trans*-encoded sRNAs, several interaction partners have been identified and biological functions in different areas elucidated. However, for the majority of the sRNAs, targets have yet to be identified and perhaps new mechanisms of action have yet to be unraveled. In addition, the recent discovery of the sponge RNA RosA [43] indicates that large networks exist that include both novel and already known regulatory RNAs.

In contrast to Gram-negative bacteria, *B. subtilis* does not seem to rely on two general RNA chaperones to promote the majority of sRNA-target RNA interactions or to stabilize sRNAs but seems to employ several RNA-binding proteins depending not only on the corresponding sRNA, but the specific sRNA/target RNA system, as illustrated by SR1/*ahrC* and SR1/*kinA*. A global approach is required to find out if the function of CsrA is confined to the SR1/*ahrC* system or if it plays a broader role in *B. subtilis* similar to Hfq and ProQ in Gram-negative bacteria like *E. coli* or *Salmonella enteric* [64]. Moreover, it has to be experimentally validated if FbpA, FbpB, and FbpC [40,58] are indeed RNA chaperones. Hitherto, only two bifunctional regulatory RNAs, SR1 and SR7, have been identified in *B. subtilis* (see Figure 3), but an increase in the number of such RNAs can be anticipated. Dual-function sRNAs and antisense RNAs comprising small ORFs that are translated under certain conditions will allow incorporating small proteins into extensive regulatory RNA–RNA networks. Beyond that, we hypothesize that sRNAs will be discovered that bind and modulate enzymes or act directly on the *Bacillus subtilis* chromosome like a number of eukaryotic siRNAs.

## Figures and Tables

**Table 1:** Overview and characteristics of all known *cis*-encoded sRNAs and their target RNAs in *Bacillus subtilis*.

sRNA	sRNA Length	Mechanism of Action of sRNA	Target RNA	Target Gene Function	Regulation, Peculiarity
RatA	222 nt	RD	*txpA*	Toxin	Glucose dependent
SR4	180 nt	RD + TI	*bsrG*	Toxin	Temperature dependent
SR5	163 nt	RD	*bsrE*	Toxin	Multistress responsive
SR6	100/215 nt *	RD TI	*yonT* *yoyJ*	Toxin Toxin	Multistress responsive
*as-bsrH*	200 nt	RD	*bsrH*	Toxin	Multistress responsive
S25	≈1350 nt	unknown	*yabE*	Autolysin	sRNA under control of σ^M^ and σ^X^
S1559	667 nt	unknown	*gdpP*	c-di-AMP PD	sRNA under σ^D^ control
S1326	700–2200 nt ^#^	unknown	*cwlO*	Autolysin	sRNA under σ^B^ control
S1290	300–3800 nt ^#^	T interference	*opuB*	Choline transporter	sRNA under σ^B^ control
SR7	185/259 nt	T interference	*rpsD*	Ribosomal protein S4	sRNA under σ^B^ control; dual-function antisense RNA

RD, promotion of RNA degradation; TI, translational inhibition; T interference, transcriptional interference; PD, phosphodiesterase; * longer SR6 species due to read-through of the SR6 terminator; ^#^ several longer RNA species due to read-through of the terminator.

**Table 2:** Overview and characteristics of all known *trans*-encoded sRNAs and their target genes in *Bacillus subtilis*.

sRNA	sRNA Length	Mechanism of Action of sRNA	Target RNA	Target Gene Function	Regulation
SR1	205 nt	TI TI	*ahrC* *kinA*	Arginine catabolism Sporulation initiation	CcpN, CcpA, sporulation
FsrA	84 nt	TI TI TI TI TI TI	*sdhCAB* *citB* *gltA* *lutABC* *dctP* *leuCD*	Iron sparing response Aconitase Glutamate synthase Iron-sulfur oxidase Dicarboxylate permease Leucine biosynthesis	Fur, iron; Some targets need FbpA, B, or C
RnaC	125 nt ?	RD + TI ?	*abrB*	Transition state regulation	Growth phase
RoxS	115 nt	TI + RD TI + RD RD RS + TA	*ppnKB* *sucC* *acsA* *yflS*	Redox regulation, TCA cycle, Acetyl-CoA synthetase Malate transporter	ResD (NO), Rex (malate)
RosA	92/128/ 193/225 nt *	Seq	RoxS FsrA	*Trans*-encoded sRNA *Trans*-encoded sRNA	CcpA

TI, translational inhibition; TA, translational activation; RD, promotion of RNA degradation; RS, RNA stabilization; Seq, sequestration of other RNAs; * several distinct RNA species were detected in Northern blotting; ? length unknown.

## Data Availability

Not applicable.
